# CARING FOR THOSE WHO CARE: EVALUATION OF A COMPREHENSIVE CULTURAL COMPETENCY TRAINING CURRICULUM

**DOI:** 10.1093/geroni/igac059.2352

**Published:** 2022-12-20

**Authors:** Sara Powers, Rachel Cannon, David Bass, Lauren Pongan, Ocean Le, Nina Darby

**Affiliations:** The Benjamin Rose Institute on Aging, Cleveland, Ohio, United States; The Benjamin Rose Institute on Aging, Cleveland, Ohio, United States; The Benjamin Rose Institute on Aging, Cleveland, Ohio, United States; Diverse Elders Coalition, New York, New York, United States; Diverse Elders Coalition, New York, New York, United States; Diverse Elders Coalition, New York, New York, United States

## Abstract

By 2030, nearly 3 in 10 older Americans will identify as a member of a diverse or underrepresented group. To better support diverse, aging communities and help professionals meet the growing needs of family and friend caregivers, the Diverse Elders Coalition developed a comprehensive cultural competency training curriculum that, in the first year of development, engaged over 2,500 healthcare and social service providers. To evaluate the short and long-term impact of the training curriculum, this paper focuses on two types of anonymous evaluations that were electronically distributed to training attendees: 1) Subjective knowledge post-tests (n=162), and 2) 3-month post-training follow-ups (n=232). Majority of participants identified as female, White/Caucasian, and earned at least a college degree. Upon completion of the trainings, participants reported improved subjective knowledge about diverse communities, more confidence and preparedness to meet diverse caregivers’ needs, and also indicated that because of the training they would engage in a variety of diversity related actions (e.g., use more inclusive language, share training resources with colleagues, attend additional diversity trainings). At the 3-month follow-up, 91.8% of respondents reported they engaged in two or more diversity related actions since attending the training. Respondents (73.8%) also indicated that their organization engaged in one or more diversity-related actions since attending the training (e.g., provided staff additional diversity-related trainings, translated materials). Discussion will focus on ways to improve healthcare and social service providers’ advocacy efforts and awareness surrounding the needs of older adults and caregivers from diverse communities.

